# Bulwer & Forbes on the Water Treatment

**Published:** 1851-07

**Authors:** 


### EDITORIAL DEPARTMENT.

*Bulwer & Forbes on the Water Treatment.* Edited, with additional matter, by ROLAND S. HOUGHTON, A. M., M. D. 12 mo., pp. 258. Fowlers & Wells publishers, Clinton Hall, 131 Nassau street.

This is a work designed for the popular reader, and prepared with a view to advance the cause of hydropathia with the public. The Editor is a hydropathist, and, we will venture to say, although he does not announce the fact, either is, or expects to be connected with some *water-cure.* To write or compile a book on some speciality in therapeutics, which shall circulate among unprofessional readers, is one of the most effective means, in these modern days, of securing patients.

The history by Bulwer of his personal experience of the effects of the water treatment, is just what might be expected from a person of his enthusiastic temperament. That he is a genius, and gifted with fine imaginative powers, does not render him peculiarly competent to analyze the effects of remedial agencies, the causes of disease, etc., even in his own case. We do not, of course, doubt the testimony of his consciousness of improved health, nor do we question his motives, but we are in no wise bound to adopt his explanation of the facts involved in his personal experience, or the deductions therefrom. The mistake is often made of supposing that persons distinguished in some of the departments of literature, are, as a matter of course, better qualified than others to reason on subjects of medical science. The opinions of literary men, if favorable to any irregular system of practice, carry considerable weight with many. The absurdity of such an idea must be obvious. The habits of thought, and exclusiveness of attention by which excellence is attained in particular intellectual pursuits, may render a person less able to judge correctly of matters without the range of their studies, than those whose minds have been less restricted by devotion to special objects, and are only known for their general information and good sense. And it is always to be observed that persons of the latter description are the unwavering friends of legitimate medicine. A novelist, the business of whose life has been to roam in the regions of fancy, is the very last person to have any claims to be considered an oracle in matters of scientific truth. The opinion of an obscure, but shrewd, intelligent mechanic would be worth vastly more. But enough of this,—Bulwer's aquatic romance is not without interest and value for the medical reader, who will receive it as he does a host of extraordinary experiences which are recounted by patients, accepting the facts, and forming his own conclusions. It is now some years since the essay was first published, long enough for it to have had its day with the public, and, probably, also with the author.

The articles by Forbes, Wilson, Scudamore, and Mayo, are well worthy of perusal. They are written in a spirit of enthusiasm, and are, therefore, doubtless somewhat excessive, that is to say, these authors anticipate more benefit from the use of water, as a remedial agent, than will probably be realized. We are free to say, however, that we think practical medicine is to be in no small measure improved by the introduction of this agent into practice to a greater extent than has hitherto been done; nor do we shrink from conceding that the peasant of Silesia, Priessnitz, has been instrumental in developing experimental facts which will subserve the cause of medical science. These facts should not be disregarded. Medicine may often thus avail herself of the efforts of the ignorant, who do not hesitate to resort to therapeutical measures that those who are better aware of the sources of danger might be restrained from venturing upon except under the sanction of precedents.

On the employment of water in the treatment of diseases, we may have something to say hereafter. Facts pertaining to this subject contributed by reliable observers are desirable.

As respects the use of water in surgery, Prof. Hamilton has rendered an useful service in translating the dissertation by Amussat, which has appeared in successive numbers of this Journal.

